# Growth Hormone Deficiency Is Associated with Worse Cardiac Function, Physical Performance, and Outcome in Chronic Heart Failure: Insights from the T.O.S.CA. GHD Study

**DOI:** 10.1371/journal.pone.0170058

**Published:** 2017-01-17

**Authors:** Michele Arcopinto, Andrea Salzano, Francesco Giallauria, Eduardo Bossone, Jörgen Isgaard, Alberto M. Marra, Emanuele Bobbio, Olga Vriz, David N. Åberg, Daniele Masarone, Amato De Paulis, Lavinia Saldamarco, Carlo Vigorito, Pietro Formisano, Massimo Niola, Francesco Perticone, Domenico Bonaduce, Luigi Saccà, Annamaria Colao, Antonio Cittadini, T.O.S.CA. (Trattamento Ormonale Scompenso CArdiaco) Investigators

**Affiliations:** 1 Dipartimento di Scienze Mediche Traslazionali, Federico II University, Naples, Italy; 2 Dipartimento di Cardiologia e Cardiochirurgia, University Hospital “Scuola Medica Salernitana”, Salerno, Italy; 3 Department of Internal Medicine, The Sahlgrenska Academy at the University of Gothenburg, Sweden; 4 IRCCS S.D.N., Naples. Italy; 5 Divisione di Cardiologia, San Daniele del Friuli Hospital, Udine, Italy; 6 Cardiologia SUN, Monaldi Hospital, AO Colli, Second University of Naples, Naples, Italy; 7 AORN "A. Cardarelli", Naples, Italy; 8 Dipartimento di Scienze Biomediche Avanzate, Federico II University, Naples, Italy; 9 Dipartimento di Scienze Mediche e Chirurgiche, Università Magna Græcia, Catanzaro, Italy; 10 Dipartimento di Medicina Clinica e Chirurgia, Divisione di Endocrinologia, Federico II University, Naples, Italy; 11 Interdisciplinary Research Centre in Biomedical Materials (CRIB), Naples, Italy; Vanderbilt University, UNITED STATES

## Abstract

**Background:**

Although mounting evidence supports the concept that growth hormone (GH) deficiency (GHD) affects cardiovascular function, no study has systematically investigated its prevalence and role in a large cohort of chronic heart failure (CHF) patients. Aim of this study is to assess the prevalence of GHD in mild-to-moderate CHF and to explore clinical and functional correlates of GHD.

**Methods:**

One-hundred thirty CHF patients underwent GH provocative test with GHRH+arginine and accordingly categorized into GH-deficiency (GHD, n = 88, age = 61.6±1.1 years, 68% men) and GH-sufficiency (GHS, n = 42, age = 63.6±1.5 years, 81% men) cohorts. Both groups received comprehensive cardiovascular examination and underwent Doppler echocardiography, cardiopulmonary exercise testing, and biochemical and hormonal assay.

**Results:**

GHD was detected in roughly 30% of CHF patients. Compared to GHD, GHS patients showed smaller end-diastolic and end-systolic LV volumes (-28%, p = .008 and -24%, p = .015, respectively), lower LV end-systolic wall stress (-21%, p = .03), higher RV performance (+18% in RV area change, p = .03), lower estimated systolic pulmonary artery pressure (-11%, p = .04), higher peak VO_2_ (+20%, p = .001) and increased ventilatory efficiency (-12% in VE/VCO_2_ slope, p = .002). After adjusting for clinical covariates (age, gender, and tertiles of LV ejection fraction, IGF-1, peak VO_2_, VE/VCO_2_ slope, and NT-proBNP), logistic multivariate analysis showed that peak VO_2_ (β = -1.92, SE = 1.67, p = .03), VE/VCO_2_ slope (β = 2.23, SE = 1.20, p = .02) and NT-proBNP (β = 2.48, SE = 1.02, p = .016), were significantly associated with GHD status. Finally, compared to GHS, GHD cohort showed higher all-cause mortality at median follow-up of 3.5 years (40% *vs*. 25%, p < .001, respectively), independent of age, sex, NT-proBNP, peak VO_2_ and LVEF.

**Conclusions:**

GH deficiency identifies a subgroup of CHF patients characterized by impaired functional capacity, LV remodeling and elevated NT-proBNP levels. GHD is also associated with increased all-cause mortality.

## Introduction

Multiple anabolic deficiencies are common in Chronic Heart Failure (CHF) and identify a subgroup of patients with worse outcome [1, 2]. Although anabolic impairment is a multifaceted phenomenon related to abnormalities of several anabolic endocrine axes, abnormalities of growth hormone/insulin-like growth factor (GH/IGF-1) physiology has long been considered a key component of the multiple hormonal deficiency syndrome of CHF. In addition, GH deficiency (GHD) *per se* is associated with increased cardiovascular mortality in the general population [3], and leads to impairment of cardiac performance [4]. Such evidence represents a solid background to test the hypothesis that CHF patients with coexisting impairment of the GH/IGF-1 axis display worse cardiovascular abnormalities compared with CHF patients with normal GH/IGF-1 status. While several studies have described IGF-1 levels in CHF with inconsistent results [2, 5], very little information is available regarding pituitary GH secretion in CHF. Assessment of prevalence and clinical relevance of GH and IGF-1 deficiency in a large population of CHF patients not only may help understanding of GH/IGF-1 physiology in CHF, but could also pave the way for innovative therapeutic approaches, such as GH replacement therapy [6, 7]. The current cross-sectional study was therefore aimed at evaluating the GH/IGF-1 status in a large population of CHF patients. Specifically, we measured GH pituitary secretion with a provocative test and assessed the relationships between hormonal parameters and clinical characteristics, exercise capacity, LV structure and function in two different cohorts of GH sufficient and deficient patients with CHF.

## Methods

### Study population

One hundred eighty-eight patients with CHF NYHA class I-III coming from 3 tertiary care centers were screened from December 2008 to December 2011 for this cross-sectional, observational trial which was registered on *ClinicalTrials*.*gov* with the code NCT00511927. Patients recruited during hospital stay were studied after a 3-month period of clinical stability. The inclusion criteria were as follows: patients of either sex affected by CHF New York Heart Association (NYHA) class I to III, secondary to ischemic or idiopathic dilated cardiomyopathy, age > 18 years, stable medications for at least three months, and LV ejection fraction 40% or less. Exclusion criteria were: active malignancy, unstable angina or recent myocardial infarction (within three months), severe liver disease, serum creatinine level > 2.5 mg/dl, and any relevant endocrine disease with the exception of diabetes. One hundred-thirty patients fullfilled the inclusion criteria and were evaluated with the following tests: Electrocardiogram (ECG), NYHA class, blood chemistry including N-terminal pro Brain Natriuretic Peptide (NT-proBNP), IGF-1, IGFBP-3, and GH stimulatory test. Patients diagnosed with GHD were subsequently studied with scan of the pituitary region. All patients underwent Doppler echocardiography and cardiopulmonary exercise stress testing. Using the Minnesota-Living with Heart Failure Questionnaire (MLHFQ) quality of life (QoL) was assessed. Anxiety and depression were assessed by a trained interviewer by using the State-Trait Anxiety Inventory [8] and the Zung Self-Rating Depression Scale [9]. GH deficiency was diagnosed according to the Guidelines of the Italian National Health Care System [10], using a Growth Hormone Releasing Hormone (GHRH) + Arginine test and BMI-adjusted cut-offs (peak GH below 9 μg/L for patients ≤ 30 kg/m^2^ BMI and 4.1 μg/L for patients with BMI > 30 kg/m^2^). Patients who performed both GH provocative test and basal IGF-1 and IGFBP-3 measurement were sampled the same day. The study fully complied with the Declaration of Helsinki and all patients signed informed consent at the enrollment. The study protocol was approved by the Ethics Committee of the Federico II University of Naples.

### Cardiopulmonary exercise stress test (CPET)

All patients underwent an incremental CPET on a bicycle ergometer. To stabilize gas measurements, patients were asked to remain still on the ergometer for at least 3 minutes before starting exercise. After a 1-minute warm-up period at 0 Watts workload, a ramp protocol of 10W/min was started and continued until exhaustion. The pedalling was kept constant at 55 to 65 revolutions/min. A 12-lead electrocardiogram (ECG) was monitored continuously during the test, and cuff blood pressure was manually recorded every 2 minutes. Respiratory gas exchange measurements were obtained breath-by-breath with use of a computerized metabolic cart (Vmax 29C Sensormedics, Yorba Linda, California, USA). Peak VO_2_ was recorded as the mean value of VO_2_ during the last 20 seconds of the test and expressed in millilitres per kilogram per minute. At the end of cardiopulmonary exercise test, patients were asked to identify the primary reason for stopping. Predicted peak VO_2_ was determined by use of a sex-, age-, height-, and weight-adjusted and protocol-specific formula outlined by Wassermann et al. [11]. The ventilatory anaerobic threshold (VAT) was detected by 2 experienced reviewers (F.G. and A.C.) by use of the V-slope method [12]. The VE versus VCO_2_ relationship was measured by plotting ventilation (VE) against carbon dioxide production (VCO_2_) obtained every 10 seconds of exercise (VE/VCO_2_ slope): both VE and VCO_2_ were measured in litres per minute. The VE/VCO_2_ slope was calculated as a linear regression function, excluding the non-linear part of the relationship after the onset of acidotic drive to ventilation.

### Echocardiography

Standardized transthoracic echocardiography and Doppler examinations were performed with Aplio XG, Toshiba (Japan). Measurements were performed on- and off-line by one experienced investigator (M.A.) and confirmed by a second echocardiography expert in a blind fashion (A.C.). Both investigators were blinded to the patients’ group allocation. All measurements were made according to European Association of Cardiovascular Imaging/American Society of Echocardiography (ASE) guidelines [13]: left ventricular (LV) mass was calculated by the Penn convention, and indexed for BSA; the LV ejection fraction (EF) was calculated by Simpson’s equation in the apical 4 and 2 chamber views. Valvular regurgitations were categorized as absent, minimal, mild, moderate, or severe. The following LV Doppler-derived diastolic measurements were measured: E and A peak velocities, E/A ratio, E wave deceleration time, isovolumic relaxation time, early (E’) diastolic velocities (mean of values from the septal and lateral corner of the mitral annulus). E/E’ ratio was assessed to estimate LV filling pressures. All reported E/E' measurements were recorded in patients with sinus rhythm. Right Ventricle (RV) dimension was measured at end-diastole from a right ventricle–focused apical 4-chamber view at three levels: basal, mid ventricular and longitudinal levels. For the assessment of RV systolic performance, Tricuspid Annular Plane Systolic Excursion (TAPSE) was measured from the lateral tricuspid annulus, and RV Fraction Area Change was calculated as the percent change in RV cavity area from end-diastole to end-systole in the apical four chamber view.

### Biochemistry

All blood samples were collected by venipuncture and the sera were centrifuged within 3 h. After centrifugation, samples were frozen and stored at -80°C until used for assay. Serum GH for stimulatory test was assayed in local laboratories with an IRMA method (Pharmacia & Upjohn Diagnostic AB, Uppsala, Sweden). Serum N-terminal prohormone brain natriuretic peptide (NT-proBNP) concentrations were measured by an electrochemiluminescence immunoassay (ECLIA by Roche diagnostics) using a Roche Elecsys analyzer. Serum concentration of total IGF-1 and IGFBP-3 were measured in an experienced core lab to assure consistency of measurements (Goteborg, Sweden). An Enzyme-Labeled Chemiluminescent Immunometric Assays (Siemens Medical Solutions Diagnostics) was used. Assays employed were The IMMULITE® 2000 IGF-I, interassay variation = 5.7 CV% and Immulite 2000 IGFBP-3, interassay variation = 3.9 CV%. IGF-1/IGFBP-3 ratio, previously employed as an index of IGF-1 peripheral activity, was calculated as reported elsewhere [5, 14]. In males, testosterone levels were also examined (DPC Coat-A-Count RIA kit).

### Statistical analysis

Continuous variables are expressed as means ± standard error of the mean (SEM). Categorical variables were expressed as percentage. All variables were tested for normal distribution using the Kolmogorov-Smirnov test. Normally distributed variables were compared between two groups using the two-sided, unpaired Student's *t*-test with the assumption of unequal variance. Non-normally distributed variables were compared between two groups using the non-parametric Mann-Whitney U-test. Skewed variables were expressed as medians with lower and upper quartiles (NT-proBNP, IGF-1 and IGFBP-3). Rates and proportions were compared between groups of interest using the chi-square test. Logistic mutivariate analysis was performed with GH status as dependent variable and age, gender, and tertiles of LV ejection fraction, IGF-1, peak VO_2_, VE/VCO_2_ slope, and NT-proBNP as covariates, chosen for their pathophysiological plausibility. Multivariable cox-regression analysis adjusted for age, sex, NT-proBNP, peak VO_2_ and LVEF was performed to determine the impact of GHD on mortality. Statistical analysis was performed using the SPSS version 16.0 package (SPSS Inc., Chicago).

## Results

Clinical parameters, blood chemistry and hormonal evaluations of CHF patients according to GH status are shown in Table 1. By using a GHRH + Arginine test and BMI-adjusted cut-offs, CHF patients were categorized as GH deficient (GHD, n = 88) and GH sufficient (GHS, n = 42). No differences in baseline anthropometric characteristics were found, with the exception of a higher prevalence of male sex in GHD (Table 1). Women of the two cohorts were all in menopause.

**Table 1:** Clinical and biochemical indexes in GH sufficient and GH deficient patients with CHF.

	GH sufficient (n = 88)	GH deficient (n = 42)	*p*
Age (years)	61.6±1.1	63.6±1.5	.41
Sex (%, male)	68	81	.03
BMI (kg/mq)	27.7±0.5	27.9±0.6	.76
NYHA functional class*	2.4 (1.8–2.8)	2.5 (1.9–2.8)	.25
Aetiology CHF: IS/NIS (%)	58/42	61/39	.07
IGF-1 (ng/mL)*	136 (106–173)	134 (97–160)	.84
IGFBP-3 (mg/L)*	3.3 (2.7–4.1)	3.2 (2.8–3.7)	.37
IGF-1/IGF-BP3 molar ratio*	152 (133–170)	156 (134.6–172)	.72
Testosterone, males (ng/mL)	401±21	458±20	.87
Haemoglobin (mg/dL)	13.8±0.3	13.8±0.3	.94
Glycaemia (mg/dL)	109.2±7.3	96.3±1.8	.09
Diabetes (%)	20.0	21.1	.40
Total Cholesterol (mg/dL)	180.3±4.4	166.4±5.9	.08
Triglycerides (mg/dL)	105.8±4.6	122.1±10.2	.09
NT-proBNP (pg/mL)*	842 (182–2510)	794 (278–4579)	.32
MLHFQ	40±4	55±4	<0.001
Anxiety score	36±3	38±3	.24
Depression score	37±2	43±2	.001
ACE-I / ARBs (%)	91	94	.81
β-blockers (%)	78	75	.82
Digoxin (%)	23	21	.63
Spironolacton (%)	45	47	.75
Diuretics (%)	81	80	.91

BMI: body mass index; IS/NIS: Ischemic/non ischemic; IGF-1: Insulin-like Growth Factor-1; IGFBP-3: Insulin-like Growth Factor Binding Protein-3; eGFR: estimated glomerular filtration rate; MLHFQ, Minnesota Living with Heart Failure Questionnaire; NT-proBNP, N-terminal prohormone brain natriuretic peptide; ACE-I / ARBs: Angiotension Converting Enzyme-Inhibitor/Angiotension Receptor Blockers. Data expressed as mean ± SEM

*data expressed as median (interquartile range).

**Fig 1:**
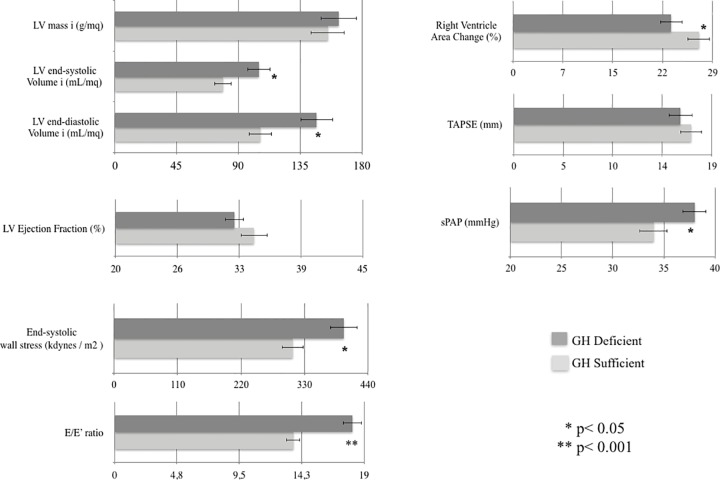
Echocardiographic indexes in GH sufficient and GH deficient patients with CHF. LV: left ventricle; TAPSE: Tricuspid Annular Plane Systolic Excursion; sPAP: systolic Pulmonary Artery Pressure.

**Fig 2:**
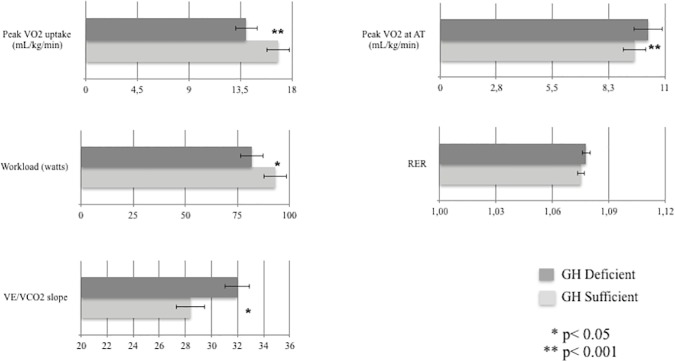
CPET parameters in GH sufficient and GH deficient patients with CHF. VO2: oxygen uptake; AT: anaerobic threshold; RER: respiratory exchange ratio; VE/VCO2: ventilation/carbon dioxide production.

GHD patients showed worse functional status as expressed by impaired cardiopulmonary and echocardiography indices (Figs 1 and 2). Compared to GHD, GHS patients showed smaller end-diastolic and end-systolic LV volumes (-28%, p = .008 and -24%, p = .015, respectively), lower LV end-systolic wall stress (-21%, p = .03), higher RV performance (+18% in RV area change, p = .03), lower estimated systolic pulmonary artery pressure (-11%, p = .04), higher peak VO_2_ (+20%, p = .001) and increased ventilatory efficiency (-12% in VE/VCO_2_ slope, p = .002). Moreover, GHD patients showed worse diastolic dysfunction as suggested by the higher E/E’ ratio (Fig 1).

**Fig 3:**
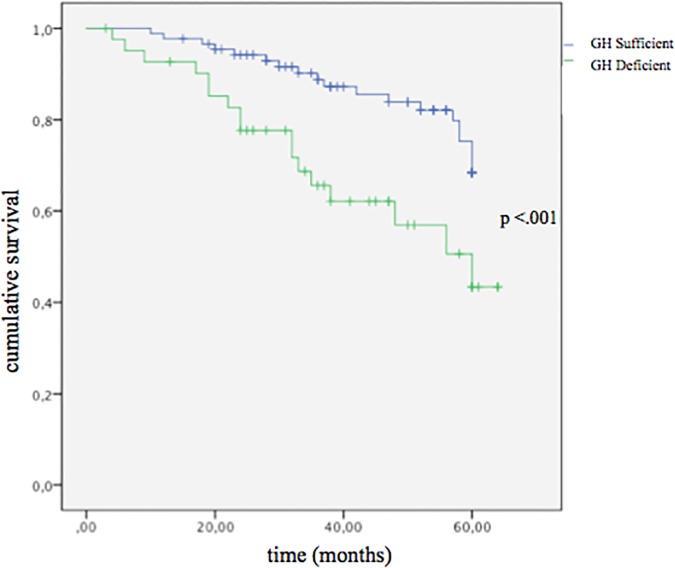
Survival analysis according to GH status: Kaplan–Meier curve and log rank analysis.

No significant differences were detected in IGF-1 and IGFBP-3 values between the two cohorts. Both groups showed similar lipid profiles and prevalence of diabetes (Table 1). Moreover, there was no significant difference in BMI; with a prevalence of obesity of 9.1% (n = 8) and 9.5% (n = 4) in GHS and GHD, respectively (p = .67). Finally, both groups were similar in terms of medication regimen (Table 1). Compared to GHS, GHD patients showed significantly worse scores of QoL (MLWHF) and Depression; whereas similar Anxiety score was found between the two cohorts (Table 1). After adjusting for clinical covariates (age, gender, and tertiles of LV ejection fraction, IGF-1, peak VO_2_, VE/VCO_2_ slope, and NT-proBNP), logistic multivariate analysis showed that peak VO_2_ (β = -1.92, SE = 1.67, p = .03), VE/VCO_2_ slope (β = 2,23, SE = 1.20, p = .02) and NT-proBNP (β = 2.48, SE = 1.02, p = .016), were significantly associated with GHD status. Finally, compared to GHS, GHD cohort showed higher all-cause mortality at median follow-up of 3.5 years (40% *vs*. 25%, p < .001, respectively) (Fig 3).

Cox-regression analysis showed that GHD cohort showed higher risk of mortality compared to GHS cohort (HR 2.11, 95%CI 1.12–4.00, p = 0.021) independent of age, sex, NT-proBNP, peak VO_2_ and LVEF (Table 2*)*.

**Table 2:** Cox-regression analysis.

	Relative Risk	95,0% CI	p value
Age	.98	[.94–1.01]	.201
Sex	.55	[.21–1.43]	.222
NT-proBNP	1.00	[.99–1.00]	.394
VO_2_ peak	.98	[.83–1.13]	.760
LVEF	.96	[.88-.03]	.232
GHD	2.11	[1.12–4.00]	.021

NT-proBNP: N-terminal prohormone brain natriuretic peptide; VO2 peak: peak oxygen consuption; LVEF: left ventricular ejection fraction; GHD: growth hormone deficiency

## Discussion

The major findings of the present cross-sectional study are: 1) GHD is common in CHF, affecting about one-third of mild-to-moderate CHF patients of our study cohort; 2) GHD identifies a subgroup of CHF with a worse clinical status, QoL and depression scores, LV remodeling, and physical performance; 3) GHD status is associated with increased NT-proBNP levels and all-cause mortality. LV structure and biventricular function in CHF were more compromised by coexistence of GHD. In particular, CHF patients with GHD displayed larger LV volumes with elevated wall stress as well as higher filling pressures as estimated by E/E’ ratio compared to patients with normal GH pituitary secretion. Right ventricle hemodynamics was also impaired in CHF patients with GHD (Fig 1). Notably, compared with GHS patients, peak VO_2_ was significantly lower in GHD patients, although similar values at anaerobic threshold were found (Fig 2). This may be secondary not only to altered myocardial mechanics, but also to the well-known GH actions on skeletal muscle, vascular bed and ventilatory dynamics. In addition, GHD-CHF patients showed reduced ventilatory efficiency as suggested by higher VE/VCO_2_ slope (Fig 2), which is an index of ventilatory response to exercise and a strong prognostic index for cardiac mortality and hospitalizations [15]. The worse LV remodeling and cardiopulmonary performance of CHF patients with GHD is not unexpected given that GHD status *per se* is associated with impaired cardiac performance, increased peripheral vascular resistance, reduced exercise capacity [16, 17]. On the other hand, CHF and GHD status are both associated with detrimental actions on skeletal muscle [18]. Although this study was not specifically designed at identifying the pathophysiological determinants of this endocrine defect, our findings support the emerging theory that the GH deficiency might be associated with indexes of neurohormonal activation. In this view, the significant association between NT-proBNP and the GHD status independently of anthropometrics, LVEF and cardiopulmonary functional capacity supports such hypothesis. The finding of an independent association between neurohormonal activation and GH axis status is supported by previous observations in patients with CHF. In the study by Niebauer et al [19], lower levels of IGF-1 (<104 ng/mL) were associated with a wide panel of cytokine and neurohormonal activation, including TNF-α, cortisol/dehydroepiandrosterone ratio, noradrenaline and adrenaline as well as a lower muscular strength. More recently, higher GH levels were found directly correlated with increasing NT-proBNP levels in a wide population of newly admitted acute HF. In this study, GH levels were independent predictors of 1-year mortality or HF readmission, and significantly improved the ADHERE risk stratification model of acute HF [20]. Circulating IGF-1 was not significantly different between GHD and GHS cohorts. Several studies have measured IGF-1 in CHF patients and in matched controls with conflicting results. Congruent with our data, Anker and colleagues reported in a similar cohort of CHF patients IGF-1 values not different from controls, [21]. In contrast, Al-Obaidi et al. [22] observed increased IGF-1 levels in mild CHF and, more recently [23], a remarkable IGF-1 increase in a large cohort of unselected CHF patients was also reported. On the other hand, several authors have reported decreased IGF-1 levels in similar CHF populations [2, 24–26]. Factors known to modulate IGF-1 levels include nutrition, level of physical activity, and CHF medications. In this regard ACE-Is and ARBs have been shown to increase IGF-1 levels in patients with CHF [27] and beta-blockers may exert a depressive action on the GH/IGF-1 axis [28]. The different (and increasing) use of such medications in the last two decades may have played a role for these discrepant results. On the other hand, it has been demonstrated that GH may influence cardiac function through IGF-1-independent mechanisms. The presence of the GH receptor gene in the myocardium (to a greater extent than in many other tissues) and vessels [29], the demonstration that GH is endowed with direct vasodilating properties [30] and the prognostic role of GH circulating levels in acute HF at 1-year [31] support such GH actions independent of IGF-1 circulating levels. To the best of our knowledge, no study has so far systematically evaluated GH/IGF-1 activity in large cohorts of CHF patients, combining measures of the IGF-1 system with stimulated pituitary responses. Broglio and colleagues were the first to report in a small group of patients with dilated cardiomyopathy (n ≈ 30) an impaired GH response to GHRH alone and combined with arginine [32]. In line with our results, Broglio and colleagues demonstrated a decreased GH secretory response in CHF patient; however, these authors reported low IGF-1 levels and no association with echocardiographic parameters. Such discrepancy might be ascribed to the small sample size and to a possible selection bias. It should be noted, in fact, that Broglio et al. enrolled CHF patients “*on waiting list for heart transplantation*”, that likely present high prevalence of GH resistance and cachexia compared to our mild-to-moderate CHF study cohort [32]. Mounting evidence suggests that deficiencies of the main anabolic axes are not mere epiphenomena but represent independent predictors of mortality in CHF [2, 33]. Our findings provide further insights into the unfavorable prognostic impact of GHD on outcome of CHF patients. Notably, patients with GHD-CHF had higher all-cause mortality rate compared with GHS (Fig 3), likely due to worse cardiopulmonary and echocardiography indexes. Larger studies are eagerly awaited in order to determine predictors of mortality in CHF patients with GHD. Interestingly, QoL and depression status, increasingly recognized as a key end point in the management of CHF have shown a significant impact on mortality in CHF [34–36]. Therefore, the observation of worse indexes of QoL and depression in GHD-CHF patients might have relevant clinical implications. Therefore, diagnosing hormonal deficiencies in CHF may lead to the identification of a subgroup of high-risk patients that may also be potentially candidate for hormonal replacement therapy. At this regard, GH replacement therapy in CHF patients determined a significant improvement of several functional indices [6, 7, 37].

*Study limitations*. The small sample size consisting of relatively young patients (mean age 62 years), predominantly men, with mild-to-moderate systolic LV dysfunction may limit our conclusions. Thus, the results may not be applicable to elderly patients or to patients with more severe LV dysfunction. In addition, the cross-sectional design of the present study does not allow us to draw mechanistic relationships between GHD and cardiovascular performance. Finally, although a more exhaustive evaluation of all neurohormonal activation indexes (i.e. plasma norephinephrine, components of the renin-angiotensin system) [38] are lacking, NT-proBNP is widely recognized as a key component of neurohormonal activation pattern [39]. Despite the afore mentioned limitations, this study has several unique strengths. In fact, this study systematically evaluated GH/IGF-1 axis activity in a large cohort of CHF patients, combining measures of systemic IGF-1 with stimulated pituitary responses.

## Conclusions

GH deficiency identifies a subgroup of CHF patients characterized by impaired functional capacity, LV remodeling and neurohormonal activation. Future prospective trials, including the ongoing TOSCA registry [40], will help to delineate the impact of anabolic deficiencies such as GHD on heart failure progression.

## Supporting Information

S1 File Minimal data set with baseline and mortality data of GHD and GHS study sub-populations. (SAV)
